# Acanthosis Nigricans in Hypochondroplasia Due to *FGFR3* Mutation

**DOI:** 10.1210/jcemcr/luaf101

**Published:** 2025-05-15

**Authors:** Joseph Reed Junkin, Victor Ramirez, Jose Bernardo Quintos

**Affiliations:** The Warren Alpert Medical School of Brown University, Providence, RI 02903, USA; Division of Pediatric Endocrinology and Diabetes, Rhode Island/Hasbro Children's Brown University Health, Providence, RI 02903, USA; Division of Pediatric Endocrinology and Diabetes, Rhode Island/Hasbro Children's Brown University Health, Providence, RI 02903, USA

**Keywords:** hypochondroplasia, short stature, acanthosis nigricans

## Image Legend

**Figure 1.**
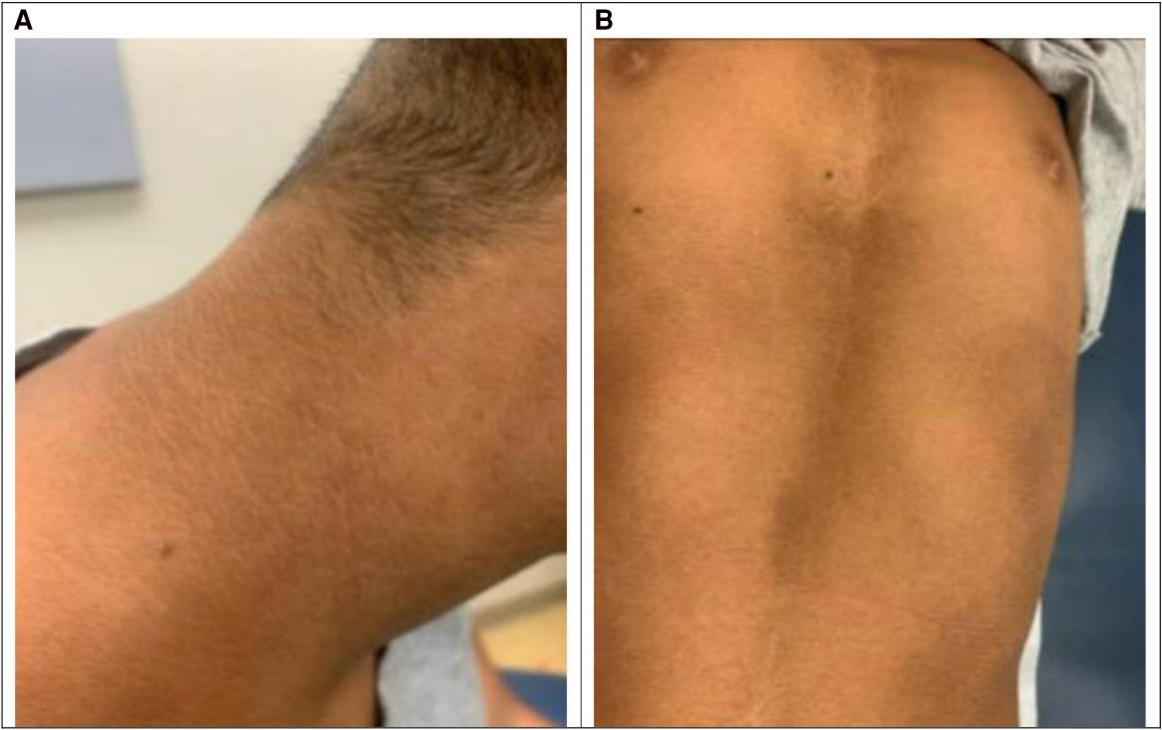
Acanthosis nigricans on the neck, abdomen and torso.

A 10-year-old boy was referred to endocrinology due to short stature. The father is 158.4 cm and mother 153.7 cm, giving their child a mid-parental height potential of 162.5 cm. On examination, the patient's height was 119.2 cm, below the first percentile (*Z* = −3.11), with weight at the second percentile (*Z* = −2.12). The upper-to-lower segment ratio was 1.27 (normal for a 10-year-old is 0.99). Screening laboratory investigations were normal for growth hormone deficiency, thyroid disease, chronic kidney disease, inflammatory bowel disease, and celiac disease. Genetic testing identified a heterozygous pathogenic *FGFR3* c.1949A > C (p.Lys650Thr) variant, associated with hypochondroplasia and acanthosis nigricans (AN) [1]. The father tested positive for the mutation, while the mother tested negative. His father has hypochondroplasia, confirming autosomal dominant transmission. Clinical images highlight AN, presenting as A, hyperpigmented, velvety patches on the posterior neck, and B, darkened skin on the medial abdomen (Fig. 1). *FGFR3* mutations primarily affect bone growth, but the same molecular mechanisms—dysregulated *FGFR3* signaling and downstream pathways—can also stimulate epidermal proliferation, distinguishing it from insulin resistance–associated AN [1, 2]. Recognizing this dermatologic manifestation is crucial for early diagnosis and genetic counseling in the setting of short stature.
